# Hyperglycemia at 1h-OGTT in Pregnancy: A Reliable Predictor of Metabolic Outcomes?

**DOI:** 10.3389/fendo.2021.612829

**Published:** 2021-05-24

**Authors:** Elena Succurro, Federica Fraticelli, Marica Franzago, Teresa Vanessa Fiorentino, Francesco Andreozzi, Ester Vitacolonna, Giorgio Sesti

**Affiliations:** ^1^ Department of Medical and Surgical Sciences, University Magna Graecia of Catanzaro, Catanzaro, Italy; ^2^ Department of Medicine and Aging, School of Medicine and Health Sciences, ‘G. d'Annunzio’ University of Chieti-Pescara, Chieti, Italy; ^3^ Department of Clinical and Molecular Medicine, University of Rome-Sapienza, Rome, Italy

**Keywords:** type 2 diabetes, cardiovascular disease, prediabetes, biomarkers, oral glucose tolerance test, hyperglycemia, gestational diabetes

## Abstract

Gestational diabetes mellitus (GDM) is associated with a high risk of developing type 2 diabetes (T2DM) and cardiovascular disease (CVD). Identifying among GDM women those who are at high risk may help prevent T2DM and, possibly CVD. Several studies have shown that in women with GDM, hyperglycemia at 1 h during an oral glucose tolerance test (OGTT) (1-h PG) is not only associated with an increase in adverse maternal and perinatal outcomes but is also an independent predictor of T2DM. Interestingly, also in pregnant women who did not meet the criteria for a GDM diagnosis, 1-h PG was an independent predictor of postpartum impaired insulin sensitivity and beta-cell dysfunction. Moreover, maternal 1- and 2-h PG levels have been found to be independently associated with insulin resistance and impaired insulin secretion also during childhood. There is evidence that hyperglycemia at 1h PG during pregnancy may identify women at high risk of future CVD, due to its association with an unfavorable CV risk profile, inflammation, arterial stiffness and endothelial dysfunction. Overall, hyperglycemia at 1h during an OGTT in pregnancy may be a valuable prediction tool for identifying women at a high risk of future T2DM, who may then benefit from therapeutic strategies aimed at preventing cardiovascular outcomes.

## Introduction

Gestational diabetes mellitus (GDM) is a clinical condition characterized by a pancreatic beta-cell dysfunction responsible for an insufficient insulin secretion that is unable to compensate for the progressive insulin resistance that characterizes the latter half of gestation (1, 2).

Women with GDM present up to seven-fold higher risk of developing type 2 diabetes (T2DM) and a two-fold greater risk of developing cardiovascular disease (CVD) than women with normoglycemia during pregnancy (3–10).

Identifying among GDM women those at high risk may help prevent T2DM and, possibly, CVD.

In the last decade, a large body of evidence has accumulated showing that the 1-h post-load glucose concentration (1-h PG) may be a useful early biomarker of dysglycemia (11). To detect specifically impaired glucose tolerance (IGT), the National Diabetes Data Group in 1979 required an interim OGTT glucose level >200 mg/dl (11.1 mmol/L) at 30, 60, or 90 min. In addition, the fasting plasma glucose (FPG) needed to be below the diagnostic threshold for diabetes (FPG >140 mg/dl [7.8 mmol/L]) and a 2-h value >140 mg/dl (7.8 mmol/L) and <200 mg/dl (11.1 mmol/L)] (12). Due to the difficulty of measuring interim glucose levels, the World Health Organization and the American Diabetes Association (ADA) abandoned the diagnostic use of the 1-h PG recommending the 2-h PG as the only post-load value required for diagnosis of IGT. Nevertheless, the measurement of 1-h PG has remained a criterion for detecting GDM.

In this review, a literature search was performed in order to examine the impact of elevated PG values during OGTT and the occurrence of T2DM in women with GDM. Furthermore, the available literature regarding the association between PG values during an OGTT in pregnancy and future cardiometabolic risk was examined (**Table 1**). 

**Table 1:** Studies evaluating cardiometabolic risk in women with hyperglycemia at 1-h during an OGTT in pregnancy.

Study	Study design	Number of participants	Geographical location	Key outcomes
Buchannan et al. (10)	Longitudinal study	91 Latino women with GDM	Los Angeles, USA	1-h PG predictor of diabetes at 1–2 years after pregnancy
Lee et al. (13)	Retrospective study	5,470 women with GDM	Melbourne, Australia	1-h PG predictor of diabetes at 15 years after pregnancy
Wein et al. (14)	Longitudinal study	2,957 women with GDM	Melbourne, Australia	1-h PG predictor of diabetes at 6 months after pregnancy
Retnakaran et al. (15)	Longitudinal study	412 pregnant women	Toronto, Canada	1-h PG predictor of prediabetes/diabetes after 3 months after pregnancy
Nishikawa et al. (16)	Retrospective study	185 women with GDM	Japan	1-h PG predictor of impaired glucose tolerance after 6–12 weeks after pregnancy
Di Cianni et al. (17)	Cross-sectional study	4,053 pregnant women	Tuscany, Italy	Independent association between 1-h PG and reduced insulin secretion
Ghio et al. (18)	Observational cross-sectional study	4,053 pregnant women	Tuscany, Italy	Progressive increase in 1-h PG levels was associated with both β-cell dysfunction and insulin resistance
Leopold et al. (19)	Longitudinal study	930 pregnant women	Vienna, Austria	Independent association between elevated 1-h PG and cord blood insulin levels and intrapartum hyperinsulinism
Retnakaran et al. (20)	Cross-sectional study	180 pregnant women	Toronto, Canada	Greater independent association between 1-h PG and hyperglycemia and insulin resistance than 2- or 3-h PG
Retnakaran et al. (21)	Cross-sectional study	361 pregnant women	Toronto, Canada	Independent association between 1-h PG and hyperglycemia, insulin resistance and β-cell dysfunction after 3 months post-partum
Scholtens et al. HAPO FUS (22)	Longitudinal study	4,832 children whose mothers were in HAPO study	International, multicentric study	Independent association between maternal glucose levels and higher glucose levels and insulin resistance during childhood
Lekva et al. (23)	Prospective cohort study	300 pregnant women	Oslo, Norway	1- and 2-h PG predictor of arterial stiffness after 5 years of follow-up
Retnakaran et al. (24)	Prospective cohort study	485 pregnant women	Toronto, Canada	1h-PG predictor of unfavorable CV profile risk after 3 months post-partum
Gungor et al. (25)	Cross-sectional study	51 pregnant women	Canakkale, Turkey	Direct correlations between 1- and 2-h PG and impaired endothelial function
Gobl et al. (26)	Longitudinal study	110 women with prior GDM	Vienna, Austria	1-h PG predictor of diabetes after 10 years after pregnancy Association between 1-h PG and subclinical inflammation and adhesion molecules
Tumminia et al. (27)	Retrospective study	297 women with GDM	Catania, Italy	1-h PG predictor post-partum 1-h OGTT ≥ 155 mg/dl

## 1h-OGTT in Pregnancy and Maternal and Neonatal Outcomes

Numerous studies have provided evidence for an independent association between PG levels during an OGTT in pregnancy and adverse maternal and neonatal outcomes (10, 28–31). In this scenario, the glucose value at 1 h during an OGTT may represent a useful early biomarker to recognize women with worse adverse outcomes.

The Hyperglycemia and Adverse Pregnancy Outcome (HAPO) study, a clinical trial conducted on a large cohort of pregnant women, showed a highly significant association between maternal fasting and post-load glucose values and increased birth weight as well as higher cord-blood serum C-peptide levels (28). Moreover, premature delivery, intensive neonatal care, and hyperbilirubinemia were significantly related to 1- and 2-h PG levels but not to FPG. Remarkably, 1-h PG was a significant independent predictor of clinical neonatal hypoglycemia (28). Similarly, in a study of 5,019 pregnant women, Kim et al. showed that women with elevated 1-h PG levels exhibited increased adverse outcomes if compared with women with normal OGTT values or women with elevated 2- and 3-h PG (29). Likewise, in pregnant Caucasian women, elevated FPG and hyperglycemia at 1 h during an OGTT were associated with a high prevalence of adverse obstetric outcomes, including caesarean delivery, macrosomia, and hypertensive disorder, whereas elevated 2- or 3-h PG did not exhibit significant differences than control group (30).

In the HAPO cohort, it has been shown that in addition to glucose levels also maternal metabolome was associated with maternal insulin resistance and newborn outcomes (31). In particular, at 1 h during an OGTT several fatty acids, triacylglycerols, amino acids, acylcarnitines, and their metabolites were associated with newborn outcomes, including adiposity and high cord C-peptide, suggesting that maternal metabolites may contribute to fetal growth and adiposity, independent of maternal BMI and glucose levels (31).

This report is in keeping with a study of 930 pregnant women, in which it has been shown that 1-h PG values between 160 and 179 mg/dl were significantly associated with increased cord-blood insulin levels, intrapartum hyperinsulinism, and macrosomia, compared to a value of 1-h PG below 160 mg/dl (19). Likewise, Mello et al. reported a significant association between 1-h PG levels and abnormal neonatal anthropometric features in pregnant women who did not meet the criteria for a GDM diagnosis (32). In particular, they found an inflection point for a threshold 1-h value of 160 mg/dl in late pregnancy in the ROC curve for the prediction of abnormal neonatal anthropometric characteristics (OR for 1-h glucose load values >160 mg/dl was 67.4 (CI, 32.1–141.2); sensitivity, 68.8%, specificity 99.02%) (32). These results are consistent with threshold of 160 mg/dl for 1-h PG values established by Weiss to define an increased risk of fetal hyperinsulinemia (33).

Overall, besides demonstrating the independent association between hyperglycemia at 1 h during an OGTT below the threshold values for the diagnosis of GDM and adverse outcomes, these data suggest that a threshold of 160 mg/dl for 1 h during OGTT may be adopted in order to identify women with an increased risk of fetal hyperinsulinemia and, consequently, at a high risk of adverse clinical outcomes. Indeed, as suggested by Desoye and Nolan (34) fetal hyperinsulinemia lowering fetal glucose levels may increase the glucose concentration gradient across the placenta and, consequently, the glucose flux to the fetus. An exaggerated glucose steal due to fetal hyperinsulinemia could also reduce maternal glucose levels and mask GDM diagnosis during OGTT (34).

## 1h-OGTT in Pregnancy and Prediction of Type 2 Diabetes

Several studies suggest that 1-h PG during an OGTT in pregnancy may be considered a relevant marker for improving metabolic risk stratification in women with GDM (10, 13–18, 20). Notably, in a longitudinal study performed on 91 Latino women with GDM, 1-h PG was the strongest independent predictor of T2DM at 1-2 years after pregnancy (10). Additionally, compared to women in the lowest tertile of 1-h PG, women in the highest tertile had a 15-fold greater risk of developing T2DM, after adjustment for insulin secretion and insulin sensitivity (10). Consistent with these data, in an analysis carried out in 5.470 women with GDM, among the glycemic parameters measured, only 1-h PG level was an independent predictor of T2DM at 15 years of follow-up (13). In particular, for each 1 mmol/L increase of 1-h PG levels, the rate of type 2 diabetes increased by 1.3 times. Neither fasting nor 2-h post-load glucose levels were found to be independent risk factors of later development of T2DM (13). Similar results were observed in a longitudinal study carried out in 2,957 women with GDM from different ethnicities, in which 1-h PG level, and not fasting or 2-h PG, was an independent predictor of postpartum T2DM (for every 1 mmol/L increase of 1-h PG level, the odds of T2DM increased by 47%) (1 h: HR, 1.3; 95% CI, 1.2–1.4; P<0.001; FPG: HR, 1.00; 95% CI, 0.9–1.1, P>0.1; 2 h: HR, 1.00; 95% CI, 0.9–1.1; P > 0.1) (14). Physiologically, the 1-h PG reflects first-phase insulin release, which is believed to be defective in GDM and type 2 diabetic patients, thus explaining its stronger predictive value in assessing the risk of T2DM development (13, 14).

Retnakaran et al. showed that in 412 pregnant women from different ethnicities, 1, 2, and 3 h post-load glucose levels were independent predictors of postpartum glucose intolerance even after adjustment for various risk factors for diabetes (1 h: OR, 1.31; 95% CI, 1.09–1.57; P = 0.0036; 2 h: OR, 1.54; 95% CI, 1.28–1.84; P<0.0001; 3 h: OR, 1.30; 95% CI, 1.09–1.55; P = 0.0030), whereas FPG was a significant predictor of only large for gestational age (LGA) (15). Furthermore, whereas FPG had the highest area under the ROC curve (AROC) for predicting LGA (0.62), the 1- and 2-h PG had the highest AROC values for postpartum prediabetes/diabetes (0.68 and 0.72, respectively), reflecting the superior discriminative capacity of post-load glucose values in the prediction of postpartum glucose intolerance (15). Similarly, in a study conducted on Japanese women, it has been shown that 1-h PG levels during an OGTT at diagnosis of GDM were significant predictors for both the need for insulin therapy in pregnancy and postpartum IGT (1 h: OR, 1.023; 95% CI, 1.009–1.037; P = 0.001; 1 h: OR, 1.027; 95% CI, 1.004–1.050; P=0.002, respectively) (16). Moreover, the cutoff value of plasma glucose levels at 1 h during an OGTT for postpartum IGT was 184.5 mg/dl (AUC, 0.770; sensitivity of 68.0%; specificity of 82.3%). On the contrary, 2-h PG levels were not found significant predictors of postpartum IGT (2 h: OR, 1.001; 95% CI, 0.980–1.023; P = 0.9) (16).

Taken together, these results suggest that elevated 1-h PG levels in women with GDM may be a valuable prediction tool for identifying women at risk for future T2DM.

## 1h-OGTT in Pregnancy and Metabolic Dysfunction

It has been suggested that among glycemic parameters, 1-h PG may reflect greater insulin resistance and beta cell dysfunction (17–21, 35). In a study involving 4.053 pregnant women, Di Cianni et al. reported that elevated 1-h PG levels were independently associated with a reduction of insulin secretion, assessed by an insulin secretion–sensitivity index, as compared with elevated FPG and 2- or 3-h PG (17). Moreover, another study conducted in the same large cohort showed that the progressive increase in 1-h PG levels was associated with a progressive loss of β-cell function as well as a decline in insulin sensitivity (18). In particular, for each 20 mg/dl increase in 1-h PG levels there was a concomitant impairment in insulin action and a reduction in insulin secretion. In addition, in women with 1-h PG <180 mg/dl, the progressive nature of defects in insulin secretion (HOMA-B: −29.7%) and insulin sensitivity (HOMA-IR: +15%) was still apparent (all P < 0.001), with a significant impairment in women with 1-h PG >160 mg/dl (18). These results were consistent with a study of 180 Caucasian, Asian, South Asian pregnant women in which elevated 1-h PG level was independently associated with greater hyperglycemia and higher insulin resistance than elevated 2- or 3-h PG (20). Furthermore, the metabolic phenotype associated with hyperglycemia at 1 h post-load resembles that of GDM, whereas that for hyperglycemia at 2 or 3 h post-load exhibits similarity to that of normotolerant women. Moreover, 1-h PG level during an OGTT was associated with lower adiponectin concentration than 2- or 3-h PG (20). Adiponectin is an adipocyte-derived hormone exerting insulin sensitizing effects whose reduction has been associated with an increased risk of T2DM (20).

Consistent with these data, Retnakaran et al. showed that elevated 1-h PG levels during pregnancy were associated with a significant metabolic dysfunction not only during pregnancy, but also at 3 months postpartum, including increased insulin resistance, and poorer β-cell function (21). Specifically, in contrast to elevated 2- or 3-h PG levels, hyperglycemia at 1 h during an OGTT bears metabolic resemblance to GDM not only during pregnancy, but also later during the postpartum period where elevated 1-h PG levels remain associated with increased glycemia, insulin resistance, and β-cell dysfunction (21). Moreover, 1-h PG levels during an OGTT was an independent negative predictor of β-cell dysfunction at 3 months postpartum (t = -3.79, P=0.0002) (21), even in pregnant women with normal glucose tolerance (35).

As recently demonstrated by an analysis of the HAPO Follow-Up Study, maternal glucose levels were associated with higher glucose levels and insulin resistance during childhood, independently of maternal and child body mass index (BMI) (22). Notably, maternal FPG was associated with child impaired fasting glucose (IFG), and 1- and 2-h PG during an OGTT in pregnancy were associated with child IGT. In addition, FPG, 1-, and 2-h PG in pregnancy were inversely associated with child insulin sensitivity, whereas 1- and 2-h PG were inversely associated with insulin secretion (22). These data suggest that children exposed in utero to higher glucose levels may be at higher risk for dysglycemic conditions associated with impaired insulin secretion and altered insulin action over time.

Moreover, in recent analyses of the HAPO Study and HAPO Follow-up Study, it has been shown that newborn adiposity is independently associated with childhood adiposity, and, along with fetal hyperinsulinemia, mediates associations of maternal glucose and BMI with childhood adiposity (36).

Taken together, these data suggest that a loss of β-cell function as well as a decline in insulin sensitivity have been found also in women with glucose levels below the threshold values for the diagnosis of GDM (18). A threshold of 160 mg/dl for 1 h during an OGTT may be useful to identify women with defects insulin sensitivity and insulin secretion who are at a high risk of postpartum metabolic dysfunction. Furthermore, this 1h-OGTT threshold may help to detect fetal hyperinsulinemia and, consequently, childhood adiposity (18, 19, 32, 33, 36).

## 1h-OGTT in Pregnancy and Cardiovascular Risk

Women with previous GDM (p-GDM) have an increased lifetime risk of developing CVD (4, 5, 8, 9). Interestingly, also in pregnant women who did not meet the criteria for a GDM diagnosis, 1-h PG was associated with an increased cardiovascular risk (24). In a prospective study, it has been shown that, among women without GDM, those with hyperglycemia at 1 h during an OGTT exhibited a worse CV risk profile at 3 months post-partum than women exhibiting elevated 2- and 3-h PG levels, suggesting that 1-h PG levels could capture an unrecognized patient population at risk for CVD (24).

Moreover, Lekva et al. found that 1- and 2-h PG levels were predictor of high pulse wave velocity (PWV) after 5 years of follow-up (23). It is known that high PWV reflects increased arterial stiffness, a strong predictor of future CV events (37). Accordingly, Gungor et al showed that both GDM and impaired glucose metabolism in pregnancy were associated with endothelial dysfunction, and that endothelial dysfunction was a significantly correlated with 1- and 2-h PG during an OGTT (25).

## 1h-OGTT in Pregnant Women After Bariatric Surgery: a Better Reliable Marker Than 2h-OGTT?

Maternal BMI represents an additional risk factor of progression to postpartum T2DM and CVD. As observed by Liu et al., pre-pregnancy BMI ≥ 30 kg/m^2^ conferred a 6.54-fold higher risk of developing T2DM, and a 1.79-fold higher risk of developing prediabetes than women with BMI< 23 kg/m^2^ (38).

It has been reported that maternal p-GDM as well as pre-pregnancy or postpartum obesity increased by 50-fold the risk of T2DM and hyperglycemia than women without obesity and GDM (39).

A large prospective cohort study showed that bariatric surgery was associated with lower risk of GDM, and large for gestational age infants, as well as with an increased risk of small-for-gestational-age infants, and, possibly, an increased stillbirth or neonatal death (40). However, the diagnosis of GDM was made according to FPG and 2-h PG levels during an OGTT, without considering 1-h PG values. The use of 2-h glucose values during an OGTT routinely performed also in other studies might be considered inappropriate to detect hyperglycemia in pregnant women after bariatric surgery (41, 42). Indeed, a history of gastric bypass surgery can influence the results of the OGTT recommended during pregnancy. Women after bariatric surgery had lower FPG levels as compared with lean, women with obesity, and BMI-matched controls, and showed altered postprandial glucose kinetics, including a rise at 1 h followed by hypoglycemia. The GDM incidence was lower in gastric bypass patients when FPG and 2-h PG levels (and not 60 min glucose levels) were used to classify hyperglycemia, while there was a markedly increased incidence of GDM when also 1-h PG levels were considered for diagnosis (42). In case of dumping syndrome or rapid gastric emptying, the conventional OGTT test should be avoided, and serial capillary glucose monitoring before and after meals should be used as an alternative, paying particular consideration to the value at 1 h after meals for a week at 24 to 28 weeks' gestation (43, 44). These data suggest that the detection of the plasma glucose value at 1 h may be a better predictive parameter for cardiometabolic outcomes in pregnant women after bariatric surgery.

## 1h-OGTT After Pregnancy and Cardiometabolic Prediction

It has been shown that hyperglycemia at 1 h during an OGTT after pregnancy may contribute to risk stratification of women with p-GDM (26). Gobl et al. reported that, in women with p-GDM, 1-h PG levels were a better predictor for insulin sensitivity and insulin secretion at 3-6 months after delivery compared to other time points during an OGTT (26). In addition, 1-h PG levels were significantly related with an increased risk of diabetes over 10 years of follow-up (HR, 1.63; 95% CI, 1.36–1.97, P < 0.001) (26).

Overall, accrued data on the impact of hyperglycemia at 1 h during an OGTT in GDM are consistent with those observed in general population suggesting that 1-h hyperglycemia during an OGTT is an independent predictor for future T2DM (11, 45–51). Indeed, in 2007 Abdul-Ghani et al. showed that PG value at 1 h during an OGTT was a better predictor for future T2DM than FPG or 2-h PG values (45). In the San Antonio Heart Study, a cutoff of 155 mg/dl for the 1-h post-load PG was successfully identified to detect a group of normal glucose-tolerant (NGT) subjects who were at risk for T2DM (46). Subsequently, these data were confirmed by several longitudinal studies in different ethnic groups as well as in a meta-analysis of prospective studies showing that NGT subjects with 1-h post-load glucose >155 mg/dl (NGT 1 h-high) have a four-fold increased risk to develop T2DM [OR 4.33 95% CI 3.40 to 5.51] (11, 47–51).

It has been reported that in women with GDM hyperglycemia at 1 h during an OGTT was an independent predictor of post-partum NGT 1h-high (27). Indeed, women with high 1h-OGTT values during pregnancy had a 3.7-fold increased risk to have high 1-h OGTT values after pregnancy than those with normal 1h-OGTT value. In addition, women with NGT 1h high exhibited an impaired insulin secretion and greater insulin resistance already during pregnancy than NGT women with 1h-OGTT low (27). Similar results have been observed in general population as several studies have shown that subjects with NGT 1h high exhibit impaired peripheral insulin action and beta-cell dysfunction (52–55). Interestingly, it has been shown that both 1- and 2-h PG levels, but not FPG, were independently associated with myocardial insulin resistance, suggesting a role of post-load hyperglycemia in determining development of CVD (55).

Additionally, it has been reported a significant association between 1-h PG levels during pregnancy and markers of subclinical systemic inflammation such as high sensitive C-reactive protein (r =0.43, P<0.001) as well as markers of endothelial dysfunction including plasminogen activator inhibitor 1 (r =0.36, P<0.001), tissue plasminogen activator (r =0.40, P <0.001), endothelial-leukocyte adhesion molecule 1 (r =0.32, P<0.001), and intercellular adhesion molecule (ICAM)-1 (r =0.37, P<0.001) (56).

In general population hyperglycemia at 1 h during an OGTT has been associated with CV subclinical organ damage, such as endothelial dysfunction, arterial stiffness, early atherosclerosis and with an unfavorable cardiometabolic risk profile, including worse lipid profile, adiposity and inflammatory markers (11, 27, 46, 50–60), an increased risk of developing CVD and all-cause mortality (51, 57). Moreover, in NGT subjects the 1-h PG concentration >155 mg/dl was independently associated with an 28% increased risk of all-cause mortality during a 33-year follow-up than subjects with low 1-h PG (57). Taken together with the data reported in pregnancy and in women with p-GDM, it is tempting to suggest that hyperglycemia at 1 h during an OGTT may identify a sizable proportion of subjects, who are at increased risk to develop T2DM, and its related cardiovascular complications.

## Conclusions

Prior literature has focused enormously on the risk of developing T2DM later in life in women with p-GDM, considering pregnancy as a possible stress test for the beta-cells (4). Over the last decades, several studies have observed the relationship between GDM and future risk of CVD, highlighting the importance of early preventive intervention, as well as the promotion of a healthy lifestyle (4, 9, 61).

Identifying among women with GDM those at high risk for adverse cardiometabolic outcomes may help prevent T2DM and its related CV complications. Examination of PG values at different times during an OGTT could provide useful information for the identification of women at high risk for T2DM.

Several studies have provided evidence for an independent predictive role of the glucose value at 1 h during an OGTT in the recognition of women at worse maternal and neonatal outcomes (19, 28–33). Interestingly, an independent association between hyperglycemia at 1 h during an OGTT and adverse maternal and perinatal outcomes has been found also in women with glucose levels below the threshold values for the diagnosis of GDM. These findings raise the possibility that adopting a lower threshold value at 1 h during OGTT (160 mg/dl) may be useful to identify women with an increased risk of fetal hyperinsulinemia and, consequently, at high risk of adverse clinical outcomes (19, 32, 33).

It has been shown that among glycemic parameters elevated 1-h PG levels may be a more sensitive prediction tool for identifying the women with GDM at risk for future T2DM, which could benefit from preventative measures (10, 13–18, 20). Additionally, hyperglycemia at 1-h PG during an OGTT may be considered a relevant biomarker for identifying pregnant women with insulin resistance, β-cell dysfunction and fetal hyperinsulinism, and with an unfavorable cardiovascular profile at post-partum (10, 17–21, 35). Moreover, maternal 1- and 2-h PG levels were also associated with child insulin resistance and impaired insulin secretion, suggesting that children exposed in utero to higher glucose levels may be at higher risk for progression to T2DM (22).

Furthermore, it has been shown that hyperglycemia at 1 h during an OGTT in pregnancy may identify women at high risk of future CVD, who may benefit from surveillance and modification of CV risk factors (23, 24, 37). These data are in agreement with robust evidence gathered in general population showing that hyperglycemia at 1 h during an OGTT is an independent predictor for T2DM, CVD, and all-cause mortality (11, 27, 46–57).

Further prospective studies with larger cohorts are needed in order to evaluate whether lifestyle changes may help these women to prevent clinical adverse outcomes and reduce the risk of future T2DM and CVD.

## Author Contributions

ES wrote and edited the manuscript. FF, MF, TF, and FA researched data and reviewed the manuscript. EV wrote and reviewed the manuscript. GS wrote and reviewed the manuscript. GS is the guarantor of the study, conceived the study, and takes full responsibility for the work. All authors contributed to the article and approved the submitted version.

## Conflict of Interest

The authors declare that the research was conducted in the absence of any commercial or financial relationships that could be construed as a potential conflict of interest.
